# Neuroinflammation increases oxygen extraction in a mouse model of Alzheimer’s disease

**DOI:** 10.1186/s13195-024-01444-5

**Published:** 2024-04-10

**Authors:** Chang Liu, Alfredo Cárdenas-Rivera, Shayna Teitelbaum, Austin Birmingham, Mohammed Alfadhel, Mohammad A. Yaseen

**Affiliations:** Department of Bioengineering, Northeastern University, Boston, MA 02115 USA

**Keywords:** Alzheimer’s disease, Neuroinflammation, Cerebral oxygenation and hemodynamics, Two-photon phosphorescence lifetime microscopy

## Abstract

**Background:**

Neuroinflammation, impaired metabolism, and hypoperfusion are fundamental pathological hallmarks of early Alzheimer’s disease (AD). Numerous studies have asserted a close association between neuroinflammation and disrupted cerebral energetics. During AD progression and other neurodegenerative disorders, a persistent state of chronic neuroinflammation reportedly exacerbates cytotoxicity and potentiates neuronal death. Here, we assessed the impact of a neuroinflammatory challenge on metabolic demand and microvascular hemodynamics in the somatosensory cortex of an AD mouse model.

**Methods:**

We utilized in vivo 2-photon microscopy and the phosphorescent oxygen sensor Oxyphor 2P to measure partial pressure of oxygen (pO2) and capillary red blood cell flux in cortical microvessels of awake mice. Intravascular pO2 and capillary RBC flux measurements were performed in 8-month-old APPswe/PS1dE9 mice and wildtype littermates on days 0, 7, and 14 of a 14-day period of lipopolysaccharide-induced neuroinflammation.

**Results:**

Before the induced inflammatory challenge, AD mice demonstrated reduced metabolic demand but similar capillary red blood cell flux as their wild type counterparts. Neuroinflammation provoked significant reductions in cerebral intravascular oxygen levels and elevated oxygen extraction in both animal groups, without significantly altering red blood cell flux in capillaries.

**Conclusions:**

This study provides evidence that neuroinflammation alters cerebral oxygen demand at the early stages of AD without substantially altering vascular oxygen supply. The results will guide our understanding of neuroinflammation’s influence on neuroimaging biomarkers for early AD diagnosis.

**Supplementary Information:**

The online version contains supplementary material available at 10.1186/s13195-024-01444-5.

## Background

Neuroinflammation is a key contributor to the pathogenesis of Alzheimer’s disease (AD) and other neurodegenerative disorders [1–4]. Activated microglia and astrocytes constitute the principal mediators of neuroinflammation through overproduction of proinflammatory cytokines, chemokines, and reactive oxygen species (ROS), while vascular endothelial cells contribute through expression of vascular and cellular adhesion molecules [5–9]. In addition to their vital roles in neuroinflammation, these cells are also crucial components of the neuro-glio-vascular unit (NGVU), which act in concert to locally regulate microvascular blood flow to satisfy the brain’s dynamic, heterogeneous energy demands [10]. Disruptions to the NGVU account for many of AD’s other preclinical hallmarks, including reductions to cerebral perfusion, impaired glucose and oxygen metabolism, and neurovascular decoupling [11–15].

A growing body of evidence highlights the causative and exacerbating roles of inflammation in the preclinical etiology of AD and other neurodegenerative disorders [2, 3, 16–20]. In diseases such as multiple sclerosis and Parkinson’s disease, a single immune stimulus can initiate a chronic neuroinflammatory phenotype in microglia and promote self-perpetuating neurotoxicity [20, 21]. In the earliest stages of AD, microglial activation reportedly precedes formation of Amyloid β plaques [22, 23]. Proinflammatory cytokines and chemokines promote synaptic loss, Aβ aggregation, increased oxidative stress, and mitochondrial dysfunction [20, 24]. Because it potentiates cerebrovascular pathology, mitochondrial dysfunction, and Aβ accumulation, the critical role of neuroinflammation in preclinical AD’s destructive positive-feedback cascade continues to gain recognition [13, 20]. Consequently, the influence of neuroinflammation on cerebral blood flow and energy metabolism warrants particular consideration [25–27]. In peripheral tissues, immunological studies have demonstrated that systemic inflammatory responses alter oxygen and glucose metabolism, mitochondrial membrane permeability and oxidative phosphorylation, and adenosine triphosphate (ATP) consumption [19, 28, 29]. Similarly, in the central nervous system, neuroinflammation stimulates production of ROS while inducing a glycolytic shift and mitochondrial fission or fusion in microglia and astrocytes, all of which significantly alters oxygen and glucose consumption and associated aerobic ATP production [28, 30–33]. Alterations to morphology, function, and metabolism of astrocytes, vascular endothelial cells, and microglia likely confound their roles in the NGVU for moderating supply of oxygen and other metabolites. The corresponding surge of neutrophils, monocytes, and lymphocytes and their associated cytokines in the bloodstream can also disrupt circulation through microvascular networks [34, 35]. Consequently, neuroinflammation directly impacts translatable biomarkers of AD progression by altering the dynamic cerebral blood flow and oxygenation signals underlying functional MRI, near-infrared spectroscopy, and positron emission tomography readouts [36, 37]. Previous neuroimaging studies reported empirical correlations between increased neuroinflammation and reduced brain energy metabolism in AD patients and in preclinical AD mouse models [26, 27], as well as studies of functional somatic syndrome and mild traumatic brain injury [38, 39]. To precisely understand how it can aggravate and accelerate clinically observable signatures of AD’s pathogenesis, the impact of neuroinflammation’s pronounced influence on cerebral energetics and hemodynamics requires investigation at the microscopic scale in live brains.

Neuroinflammatory processes reportedly exert both beneficial and harmful effects during disease progression [1, 40]. These double-edged effects have motivated several studies exploring the potential effects of enhancing or repressing a pro-inflammatory response [41]. In this study, we investigated whether changes in cortical oxygenation, microvascular hemodynamics, and neuroinflammation are evident at the early stages of AD progression in an AD mouse model. We then explored the presumptive catalytic role of a pronounced neuroinflammatory stimulus on neurovascular and metabolic dysregulation in AD progression. We applied in vivo two-photon phosphorescence lifetime imaging microscopy (2P-PLIM) with the novel oxygen-sensitive sensor, Oxyphor 2P, to measure oxygen partial pressure (pO2) and capillary red blood cell flux (RBC flux) in awake APPswe/PS1dE9 mice and wildtype controls at age 7–8 months [42–44]. The 2P-PLIM method uniquely enables nondisruptive, longitudinal characterization of pO2 and cerebral hemodynamics within individual microvessels. Previously, two-photon microscopy has revealed widely-varying neuronal and microvascular densities across different cortical layers, supporting observed laminar variations of cortical energy metabolism [45–50]. Capitalizing on the technique’s penetration depth and unparalleled spatial resolution, we quantified layer-specific differences in vascular oxygen extraction and capillary flux provoked by a prolonged inflammatory threat. Microvascular pO2 and capillary RBC flux measurements were collected from cortical layers I-IV before, during, and after 14 days pro-inflammatory stimulus, induced by daily intraperitoneal injection of the bacterial endotoxin Lipopolysaccharide (LPS) [51–55]. The measurements were used to compute layer-specific changes in oxygen extraction and capillary red blood cell flux. Before induced inflammation, AD mice show lower amounts of oxygen extraction compared to WT mice, reflecting reduced metabolic demand in AD brain tissue. LPS-induced inflammation provoked increased cytokine expression from microglia and astrocytes and substantially increased oxygen extraction at all cortical depths in both AD and control groups without altering RBC flux dynamics. Our results show that cerebral oxygen consumption is affected more significantly than vascular oxygen supply at early stages of AD, consistent with prior studies. A pro inflammatory stimulus increases oxygen extraction in the brain tissue without significantly altering vascular oxygen supply in the early stages of preclinical AD. The study emphasizes the interdependent nature of metabolic and neuroimmune alterations that transpire during preclinical AD.

## Methods

### Experimental design

All experiments were performed in accordance with ARRIVE guidelines for animal care, under a protocol approved by the Northeastern University Institutional Animal Care and Use Committee. Female APP/PS1dE9 mice and their age-matched wild-type littermates are used in this study (*n* = 10 each, The Jackson Laboratory, MMRRC Strain # 034829-JAX). The double transgenic AD mouse co-expresses the Swedish mutation of amyloid precursor protein (APP) and a mutant human presenilin 1 gene (PS1-dE9) and accumulates beta-amyloid deposits in the cortex at 6 months [56]. Mice were prepared with cranial window surgery at 6 months of age followed by habituation training of head-fixed restraint. Prior to LPS-induced systemic inflammation, “baseline” cerebral intravascular oxygenation and hemodynamics were measured in AD (8.19 months old on average) and WT (7.95 months old on average) mice. To induce systemic inflammation, 0.25 to 1 mg/kg, 1 mg/ml PBS-LPS solution (LPS from Escherichia coli O55:B5, Product Number: L4005, CAS-No. 93572-42-0, Sigma Aldrich) was administered to all mice via intraperitoneal injection daily for two weeks. Animals’ body weights and sickness behavior (i.e., posture, activity) were monitored daily to assess the sickness of the animal as well as the efficacy of the LPS. Two AD and three WT mice had gasping and low body temperature after the first two to three injections of LPS and did not survive LPS administration, and thus were excluded from the analyses. Microvascular pO2 and capillary RBC flux were measured after one week and two weeks of LPS injection. Following these measurements, mice were euthanized and brains (*n* = 3 in each cohort) were harvested for immunohistochemical analysis.

### Animal preparation

Following a procedure described previously [57], mice underwent cranial window surgery at age 6 months. Succinctly, ∼ 4 h before the surgery, the mouse was given IP injection of dexamethasone (4.8 mg/kg at 4 mg/ml) and cefazolin (0.5 g/kg at 200 mg/ml) to decrease brain inflammation and edema during and after surgery. During the surgery, the mouse was anesthetized with isoflurane (3 ∼ 4% for induction and 1 ∼ 2% for maintenance). Mouse head was secured on a stereotaxic instrument (David Kopf Instruments, CA, USA), and a heating pad (Harvard Apparatus, MA, USA) was placed under the mouse body to maintain physiological temperature. Hair and skin were removed to expose the skull. Using a dental drill, a 3-mm-diameter cranial window was made over the left somatosensory cortex (ML: -3 mm, AP: -2 mm to bregma). The skull was removed and the dura was left intact. A custom-made glass coverslip plug (OD = 5 mm, ID = 3 mm) was placed on top of the exposed dura and was sealed with superglue [57]. A customized head post was glued to the right hemisphere with Loctite 401. The skull was sealed with dental acrylic. To prevent dehydration, 100 µl, 5% dextrose sodium solution was injected subcutaneously. The mouse was singly housed and supported with antibiotics (40/8 mg/ml, sulfametoxazole/trimethiporim (SMX-TMP) and 50 mg/ml, carprofen in drinking water) continuously for five days for post-operative recovery and to minimize surgical infection. Buprenorphine (0.05 mg/kg at 0.03 mg/ml) was administered subcutaneously as analgesic. Headpost restraint training started at 7 days post-surgery using a custom-made imaging cradle. During training, animals were conditioned to tolerate head immobilization for durations lasting from 5 min to 2 h to get the animal acclimated to the two-photon imaging experiments. Milk was given as a reward during the training. Imaging took place one month after the surgery to ensure full recovery from surgery-induced inflammatory response [58, 59].

### Cerebral intravascular pO2 measurements

Approximately 30 min before imaging experiments, the oxygen-sensitive phosphorescent probe, Oxyphor 2P [43], was administered to mice via retro-orbital injection (80 µM, 50–60 µl) under anesthesia (∼ 2% isoflurane). The probe uniformly distributes inside the vasculature and labels blood plasma, allowing 2–3 hours’ intravascular imaging. The probe has a maximum two-photon absorption at λ = 950 nm and emits red-shifted photons with a central wavelength of 758 nm [43]. After recovering from anesthesia, the mouse was positioned in a customized cradle with its head fixed and cranial window surface leveled. Condensed milk was provided every 30 min.

**Fig. 1:**
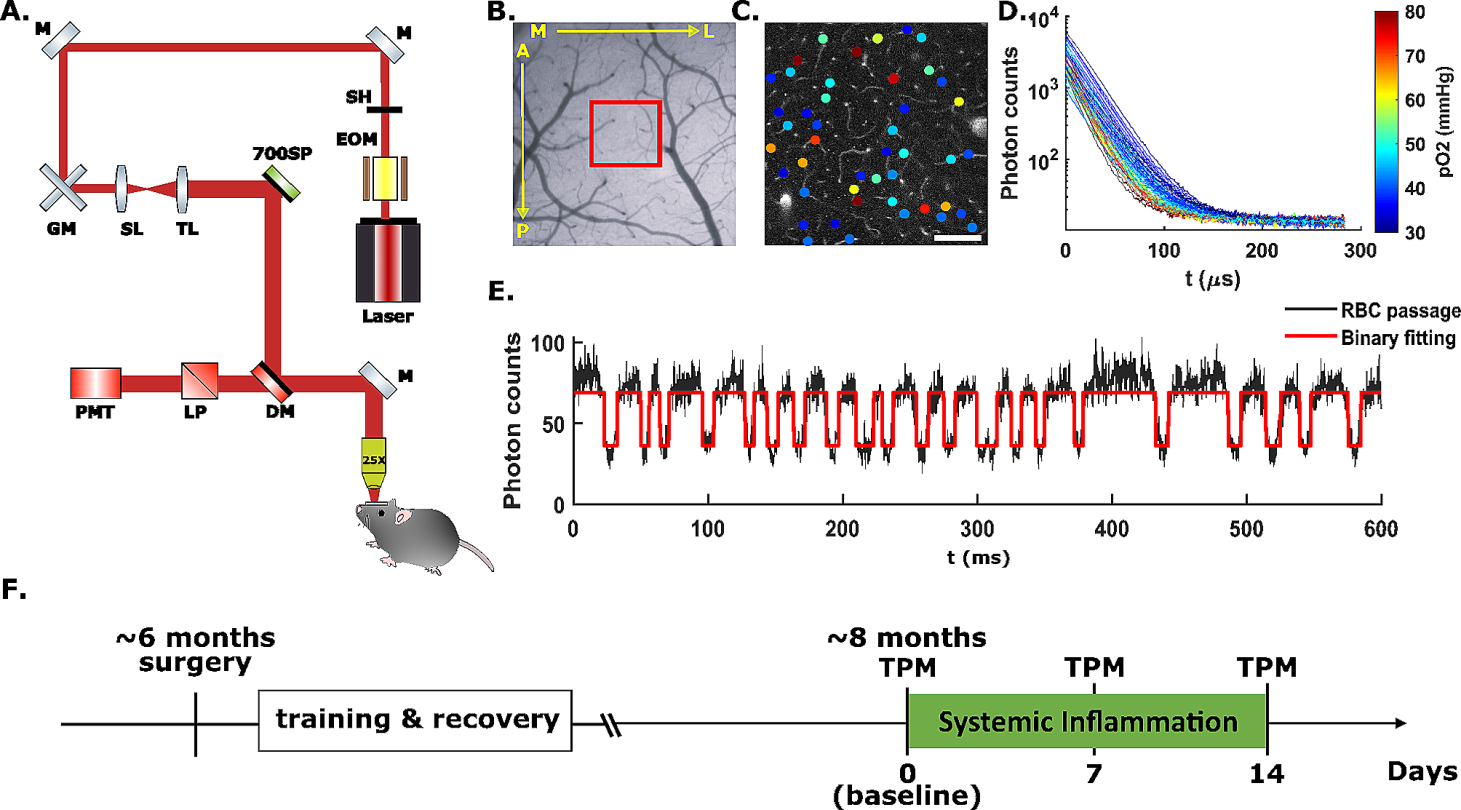
Experimental design and data acquisition. (**A**) Imaging set up for 2P-PLIM measurement in head-fixed, awake mice. 80 MHz, ultrafast pulsed laser tuned to 950 nm. The excitation laser is reflected by a 700 nm short pass filter and a 900 nm dichroic filter and is focused to the imaging field through a 25X heated, water-immersive objective. The emission light is reflected and transmitted through the dichroic and the 640 long-pass emission filter, and then detected by a photon-counting PMT. (**B**) Wide-field imaging of an AD mouse cranial window in the left somatosensory cortex. The red box indicates the region (Field of View: 474.27 × 474.27 µm^2^) being measured by two-photon microscopy. A: anterior, P: posterior, M: medial, L: lateral. **C** Two-photon survey scan image of the red-boxed field of view in **B** at cortical depth z = 200 μm, scale bar = 100 μm. Colored dots are points of measurement of intravascular pO2 and RBC flux, color-coded by pO2 values. **D** Time-resolved phosphorescence decays of the corresponding pixels in **C**. Decays are color-coded by pO2 values. **E** Time-integrated phosphorescence intensity within a single capillary measurement over 600 ms (corresponding to 2000 PLIM measurements). Black curve is the time-integrated photon total counts. Local minima correspond to single, non-labelled erythrocytes traversing through the capillary (black). The black photon count trace was thresholded (red) to count the number of RBCs (valleys of the intensity curve) over time. **F** Experimental timeline. EOM: electro-optic modulator. SH: shutter. M: mirror. GM: galvanometer scanner. SL: scan lens. TL: tube lens. SP: short-pass filter. LP: long-pass filter. DM: dichroic mirror. PMT: GaAsP photomultiplier tube

Phosphorescence lifetime measurements were performed using a commercial two-photon microscope (Ultima2P plus, BrukerNano, Inc.), a tunable ultrafast pulsed laser (InSightX3+, Spectral-Physics, US) with a repetition rate of 80 MHz was tuned at 950 nm, and a water-immersion 25X objective (N25X-APO-MP − 25X Nikon, 1.10 NA, 2.0 mm WD) (Fig. 1A). The objective was heated to ∼ 36.6 °C with an objective heater and temperature controller (ALA Scientific Instruments, Inc.) to prevent heat loss from the cranial window and to eliminate temperature-related variability in phosphorescence lifetime [43, 60]. For each animal, intravascular pO2 within a ~ 500 × 500 µm^2^ field of view (Fig. 1B, red box) was measured at multiple depths in the cortex. At the beginning of the experiment, the FOV was raster-scanned for 7 to 15 s, and the photon emitted from each pixel after each pulsed laser was counted through time-correlated single-photon counting (TCSPC) [61]. Emitted photons from each pixel were summed to generate a 2D image revealing the vasculature of the brain cortex (Fig. 1C). Intravascular locations for 2P-PLIM measurements and pO2 quantitation were selected based on the 2D image. Each pointwise PLIM measurement cycle consisted of a 10 µs-long excitation pulse, followed by a 290-µs-long collection of the emitted photons via time-correlated single photon counting (TCSPC). The 300-us cycle was repeated 2000 times (∼ 0.6s). The TCSPC hardware (SPC-150 N, Becker & Hickl, GmbH), accumulated a time-binned histogram of emitted photons over the 290 µs decay period, yielding an exponential distribution of the emitted photons with respect to the starting time of the excitation pulse [61]. Histograms were collected from the 2000 cycles to yield an exponential decay with high signal-to-noise ratio (SNR), allowing for accurate lifetime calculation via nonlinear curve fitting. The 2000 cycles were iterated 20 times in all selected measurement locations, such that 20 phosphorescence decays were acquired for each intravascular location. For each intravascular location, the 20 phosphorescence decays were averaged to get the mean phosphorescence decay before lifetime and pO2 calculation. Figure 1D shows the pO2 color-coded mean phosphorescence decays measured inside capillary locations in Fig. 1C.

We adhered to previous definition of cortical layer when presenting layer-specific pO2 values [44]: cortical layer I: subpial depth of z = 0 ∼ 100 μm, layer II/III: subpial depth of z = 100 ∼ 320 μm, and layer IV: subpial depth of z = 320 ∼ 450 μm. In each mouse, pO2 in diving arteriole and venules, and capillaries were measured at subpial depth of z = 50, 100, 200, 300, and 400 μm. Additional file 1: Supplementary Table 1 shows the number of arterioles and venules being measured for pO2 in both animal cohorts.

### Cerebral capillary red blood cell flux measurements

Red blood cell flux was measured within capillaries using the time-binned phosphorescence photons of the 2P-PLIM pO2 measurements. Because RBCs do not get labeled by the Oxyphor2P probe, the measured phosphorescence signal decreases as individual RBCs pass through the focal point in capillaries. Consequently, the time-resolved phosphorescence intensity trace features “valleys” representing the passage of individual erythrocytes [62]. With 2P-PLIM, phosphorescence emission photons of each 300 us cycle were binned to get the phosphorescence intensity. With 2000 cycles, a 0.6-s-long phosphorescence intensity time course I(t), (t = 0 to 0.6 s, dt = 300 us) was obtained (Fig. 1E). For each capillary segment, the mean RBC flux over all repetitions was calculated. Then the mean RBC flux of all measured capillary segments at all imaging depths were grouped into cortical layers, as described previously [44, 45]. The average RBC flux, and coefficient of variation (CV) in each cortical layer were calculated. The layer-specific results were averaged within animal cohorts to obtain the cortical layer-dependent mean RBC flux in AD and WT mice. For two WT mice, baseline RBC flux values were calculated using fluorescence measurements of dextran-conjugated FITC (70 kDa, Sigma Aldrich) on a separate day. Additional file 1: Supplementary Table 2 shows the number of capillaries measured for RBC flux for both animal cohort. Figure 1F shows the experimental timeline.

### Calculation of pO2, SO2 and coefficient of variation

Intravascular pO2 was calculated in each measurement location from the computed lifetimes of the measured phosphorescence decays. For each measured location, the mean phosphorescence decay was calculated by averaging over the 20 repetitions. In accordance with the established analysis protocol for Oxyphor 2P [43], the first 5 µs of the recorded PLIM decays were discarded. The remaining 285 µs of the averaged phosphorescence decay was fitted using a mono-exponential decay function $y\left(t\right)=A*exp\left(-\frac{t}{\tau }\right)+C$, and the decay rate $\tau$ represents the phosphorescence lifetime. pO2 was calculated from τ using a calibration equation similar to the Stern-Volmer relation [43]. In some experiments, abnormal phosphorescence decays occur due to animal motions. To exclude these instances, the total photon of each phosphorescence decay was calculated and the decay curves with outlying total photons were rejected using Grubb’s test [63] (MATLAB) before calculating the mean phosphorescence decay of the measured point. In each mouse, intravascular pO2s were grouped into cortical layers based on subsurface measurement depth. At each cortical layer, mean pO2 and the coefficient of variation (CV) were calculated. Layer-dependent pO2s and CVs were then averaged over all measured animal samples in each group (AD & WT) to obtain the mean pO2s and CVs of AD and WT animals. pO2 was converted into oxygen saturation of hemoglobin (SO2) using the Hill equation with coefficients for C57BL/6 mice (h=2.59, P50 = 40.2 mmHg) [64]. Layer-dependent SO2 for AD and WT were calculated using the same steps described in the calculation of layer-dependent pO2.

### Calculation of cortical depth-dependent oxygen extraction (DOE)

The amount of oxygen extracted from the cerebral capillary bed was estimated using the cortical depth-dependent oxygen extraction (DOE, %). In each cortical layer, the mean arteriole SO2 and venule SO2 was calculated through averaging over all measured arteriole and venule SO2s, and the oxygen extraction of the corresponding layer was calculated as the difference between the arteriole SaO2 and venule SvO2. DOE over all animals were averaged.

Cerebral oxygen extraction fraction (OEF) reflects the intricate balance between brain tissue’s metabolic demand and metabolite supply from the cerebral vasculature. This metric is used extensively in clinical neuroimaging studies as a biomarker for cognitive impairment. OEF is generally calculated as the arteriovenous difference in SO2, normalized by the arterial SO2. Typically, the arterial SO2 is considered constant at ∼ 98%, indicating complete saturation of O2 to RBCs. Because we observed significant reductions in arterial SO2 after inflammation, we opt to present our data as depth-dependent oxygen extraction, which indicates the non-normalized AV difference in SO2.

### Vessel identification

During imaging experiments, large pial vessels were traced along the penetrating path from the pial surface to the deepest visible imaging plane for arteriole and venule pO2 measurements. Arterioles and venules were distinguished later using the pO2 values. According to the values reported in awake and anesthetized animals [46, 65], we classified vessels with surface pO2 (measured at 50 *µm* below the pial surface) higher than 66 mmHg as arterioles, and those below 66 mmHg as venules. Note that the arterioles and venules were determined using the baseline pO2 values (pre LPS-induced inflammation). Capillaries were identified based on morphology and tortuosity. In each imaging plane, all visible capillary segments were selected for pO2 and RBC flux measurement. Primary branches from the penetrating vessels were considered precapillary arterioles or postcapillary venules and were excluded from the capillary pO2 calculation during data analysis.

### Tissue collection and immunofluorescence staining

After the last imaging session, a subgroup of AD and WT mice (*n* = 3 in each cohort) were intracardially perfused with heparin-dissolved 1X PBS followed by 4% paraformaldehyde (PFA). Brains were collected and stored in 4% PFA at 4 °C. We also collected the brains of age-matched AD and WT mouse that never received LPS as the control group (*n* = 1 in each cohort). The cerebrums were sectioned into 3 to 4 coronal slices (∼ 2 mm thickness) and stored in 75% ethanol. The sections were processed and stained with immunofluorescent antibodies by Dana-Farber/Harvard Cancer Center Specialized Histopathology Services (SHS) Core (Boston, MA.). Briefly, nuclei were stained with DAPI. Microglia and astrocytes were stained with Iba1-conjugated Alexa Fluor 594 and GFAP-conjugated Alexa Fluor 488, respectively. The stained brain slices were mounted on the glass slide for microscopic imaging.

### Confocal imaging and immunofluorescence analysis

Harvested, stained brain slices were imaged with a laser scanning confocal microscopy (Zeiss, LSM, 800) using a 10X objective. A 709 × 709 µm^2^ region of interest (ROI) around the somatosensory cortex in both hemispheres were selected. Two to six ROIs from 3 brain slices were imaged for each mouse. DAPI, Alexa Fluor 488, and Alexa 594 were excited with laser wavelength at 405 nm, 488 nm, and 561 nm. Pinhole sizes were set at 1 airy disk unit for each wavelength. The excitation power and detector gain were determined and fixed for all slices using one of the brightest slices to avoid intensity saturation. Z stack images were collected with a thickness of 40 μm and a step size of 3.96 μm. To quantify the expression of GFAP and Iba-1 in astrocytes and microglia cells, respectively, we conducted densitometry analysis. Maximum intensity projection (MIP) was calculated over the full 40 μm tissue thickness. The MIP images were binarized using an intensity threshold determined by the intensity histogram. Identical thresholding values for GFAP and Iba-1 were used among all MIP images for accurate calculation of pixel total intensity. The MIP images were divided based on cortical layers, using the same definition of cortical layers during two photon imaging experiment. For each cortical layer, percentage area (PA) of GFAP and Iba-1 positive cells were calculated as the ratio between the number of pixels with the intensity above the threshold to the total number of pixels.

### Pulse oximetry measurement of systemic oxygen saturation (SpO2)

The influence of LPS-induced inflammation on systemic arterial blood oxygen saturation was monitored in a separate group of APPswe/PS1dE9 and wildtype mice (*n* = 3 each, at 6 months). The same LPS-administration protocol was applied (0.03 mg/kg LPS, injected intraperitoneally daily for 14 days). On each day, SpO2 and heart rate was measured on the mouse thigh using a commercial oximetry device (MouseOX Plus, Starr Life Science). Hair was removed to reduce measurement noise. During these measurements, the mice were placed under light anesthesia (1 ∼ 1.5% isoflurane) and kept warm with a heating pad. SpO2 were recorded for 20 s to 1 min. The time-averaged readout was used to represent systemic oxygenation on the measured day.

### Statistical analysis

All statistical analyses were performed using the Statistics toolbox from MATLAB. Two-tailed, independent student’s t-test was used to identify significant differences between AD and WT groups. Lillie’s test was used to evaluate the normality assumption. Bartlett’s test was used to check the equal variance assumption. Unequal variance was corrected with Satterthwaite’s approximation included in the “ttest2” function in MATLAB. Statistical significance level was set at *α* = 0.05. One-way ANOVA with Tukey’s ‘hsd’ test was used for checking the difference between the cortical layers within each cohort. One-way repeated measures ANOVA with Bonferroni correction was used to analyze the effect of LPS-induced inflammation [66]. Sphericity was checked using Mauchly’s test. Violation of sphericity was estimated by epsilon. Statistical significance level of ANOVA was set at 0.05. *P < 0.05, **P < 0.01, *** *P* < 0.001. Sample size was determined using power analysis, assuming a 30% decrease of the measured physiological parameters (e.g., pO2) after induced inflammation, and an effect size of 0.8. *α* = 0.05.

## Results

We determined whether APPswe/PS1dE9 mice show differences in cortical oxygenation, microvascular hemodynamics, and neuroinflammation are evident at the early stages of AD progression using in vivo two-photon microscopy (TPM). We then explored how a 14-day LPS-induced neuroinflammatory stimulus alters neurovascular and metabolic regulation in AD progression.

### Arteriolar oxygen extraction in AD mice under baseline conditions

**Fig. 2:**
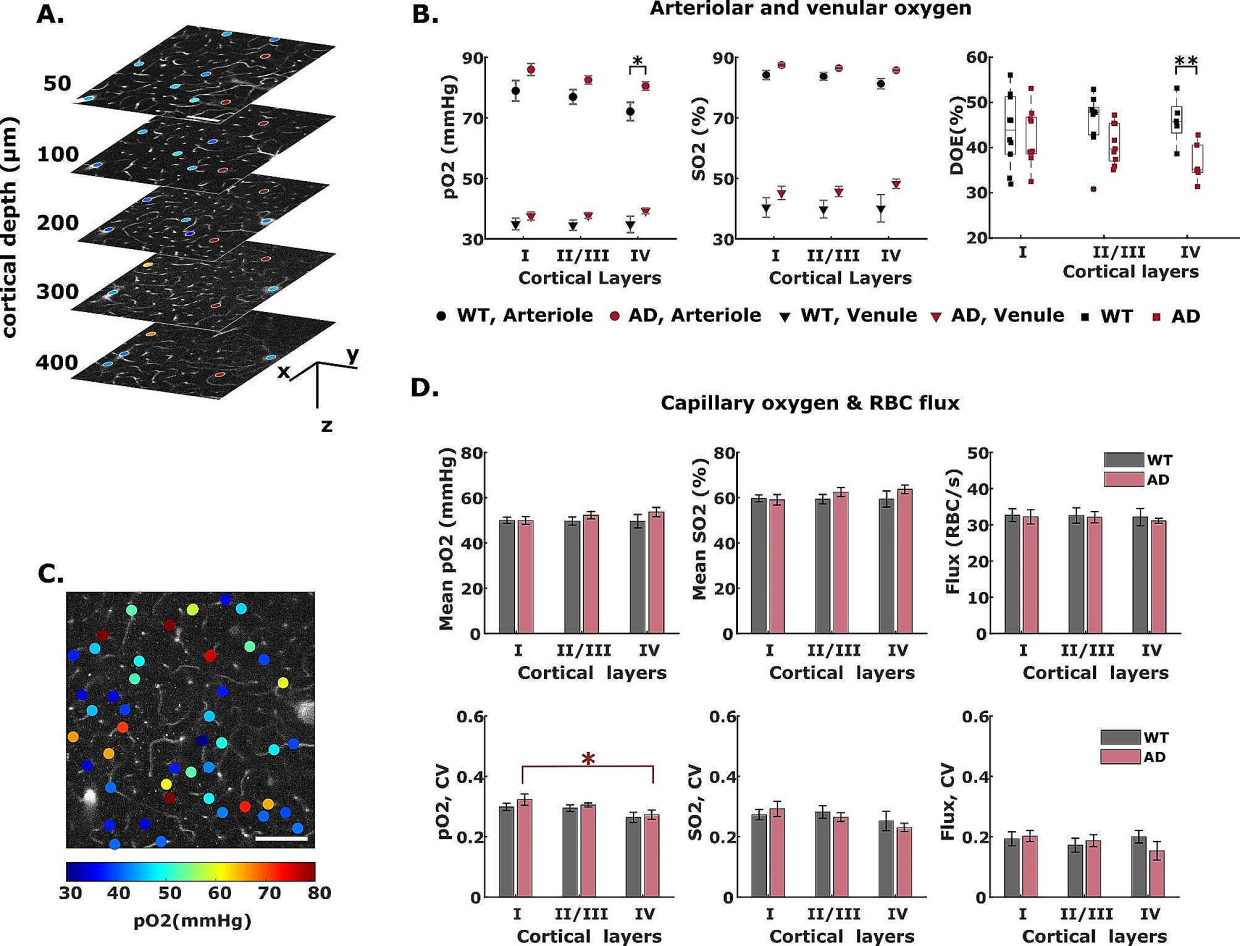
Baseline (pre LPS administration) measurement of cortical intravascular oxygenation and capillary flux in AD and WT mice. **A** 2D survey scan images of pO2 measurements in diving vessels at 50 μm to 400 μm below the pial surface (z = 0 μm). scale bar = 100 μm. Colored dots are pO2-coded measurement points. **B** Cortical-layer dependent arteriole and venule pO2 and SO2, and cortical depth-dependent oxygen extraction (DOE) in AD and WT mice. Black asterisk symbol reveals the difference between the two cohorts of animals in cortical layer IV. **C** An example survey scan image of capillary pO2 measurement at cortical depth z = 200 μm. Colored dots are pO2-coded measurement points. Colorbar is shared by **A** and **C**. **D** capillary oxygen and RBC flux. Upper row: cortical-layer-dependent capillary mean-pO2, SO2, and red blood cell (RBC) flux in AD and WT mice. Lower row: coefficient of variation (CV) of capillary pO2, SO2 and RBC flux of AD and WT mice. Data shown as mean ± standard error

Prior to an LPS-induced inflammation (aka “baseline” conditions), we measured pO2 in diving arterioles and ascending venules from layers I to IV in cortices of awake AD and WT mice at age 8 months (Fig. 2A). Our results showed that AD mice brains have slightly higher arteriolar and venular pO2 and SO2 compared to WT mice in all cortical layers (Fig. 2B). Statistically significant difference in pO2 was observed in arterioles at cortical layer IV, where the average pO2 in WT and AD is 69.33 ± 4.95 mmHg and 80.79 ± 1.65 mmHg, respectively. In cortical layer IV of AD mice brain, the comparable arteriolar SO2 but higher venular SO2 yielded significantly reduced depth-dependent oxygen extraction (DOE) in AD mice (Fig. 2B, *P* = 0.0092 in layer IV). Numerous studies indicate that maximal oxygen demand occurs in cortical layer IV, which features the highest density of mitochondria as well as cytochrome oxidase [67, 68]. Our results of reduced oxygen extraction in layer IV are indicative of diminished oxygen consumption by layer IV neurons in the presence of Amyloid β. Consistent with previous studies in awake 3–5-month-old female C57BL/6 mice [44, 69], our measurements in WT mice showed lower arteriolar and venular pO2 at deeper cortical layers, which yields a higher oxygen extraction (DOE) in deep cortical layer IV (Fig. 2B).

### Baseline capillary pO2 and RBC flux are similar in AD and WT mice

Capillary pO2 and RBC flux were measured under baseline conditions at multiple cortical depths in AD and WT mice. Similar to the findings in arteriole and venule compartments, capillary pO2 and SO2 were slightly higher in AD mice compared to WT, without reaching statistical significance (Fig. 2D). Although our capillary pO2 observations in WT mice brains are slightly higher compared to previously reported values in C57BL/6J mice (48.59 ± 0. 31 mmHg vs. 45.6 ± 1.4 in [44]), the differences are likely due to the different mouse strain and age. We calculated the coefficient of variation (CV) to evaluate the heterogeneity of capillary oxygen distribution (Fig. 2D). Consistent with previous findings by Li et al. [44], we observed progressively lower oxygenation heterogeneity (indicated by lower CV) at deeper cortical layers in both WT and AD groups (Fig. 2D), with no appreciable difference between the two cohorts. Prior theoretical studies indicate that lower heterogeneity can promote oxygen extraction and maintain adequate oxygen supply to tissue [70]. The decreased pO2 heterogeneity along cortical layers replicated previous results of more homogeneous oxygenation at deeper cortical layers in awake healthy mice [44].

We observed no significant differences between AD and WT mice in capillary RBC flux in any cortical layers (Fig. 2D), which agrees well with previous observations in a 6-month-old AD mouse model [71, 72]. RBC flux heterogeneity was also found to be similar between AD and WT mice (Fig. 2D). While decreased capillary flow and lack of capillary flow homogenization during functional activation has been observed in 18-month-old APPswe/PS1dE9 transgenic mouse [73], our results suggest that microcirculation is not profoundly altered at earlier ages.

### Neuroinflammation increases oxygen extraction in AD mice

**Fig. 3:**
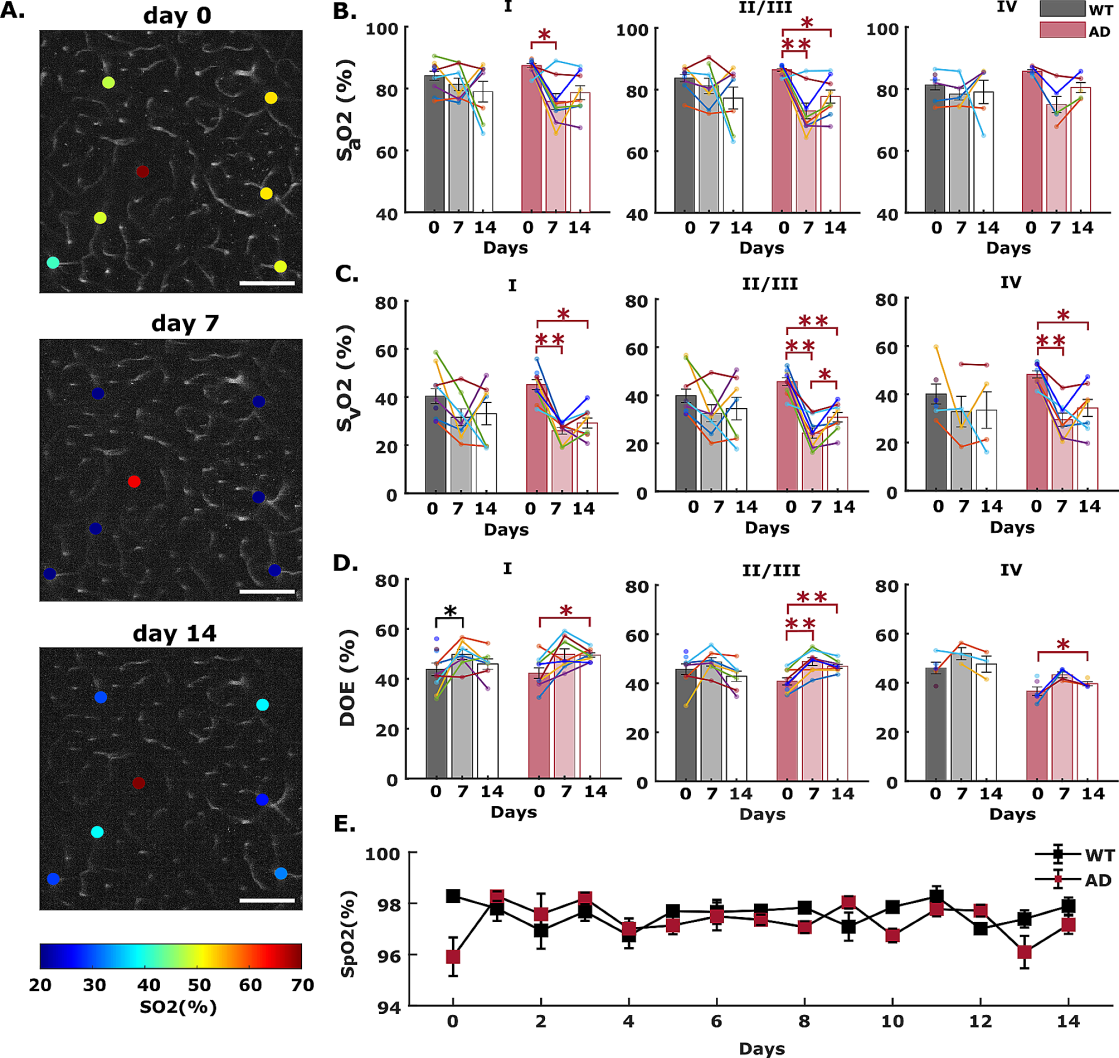
Inflammation-induced arteriole and venule oxygen reductions in WT and AD mice brain. (**A**) Example survey scan images overlaid with SO2 values in measured penetrating vessels at z = 200 μm taken on day 0 (before LPS injection), day 7 and day 14 (with daily LPS administration) in an AD mouse brain. Scale bar = 100 μm. (**B-C**) Arteriole and venule SO2 measured on day 0, and day 7 and day 14 in WT and AD mice at cortical layer I, II/III, and IV. Each bar represents mean ± standard error over all measured mice in each cohort. Connected scatterplot shows the SO2 of each individual mouse. (**D**) Depth-dependent oxygen extraction (DOE) of WT and AD mice on day 0, day 7 and day 14. (**E**) Systemic oxygen saturation measurements during a neuroinflammatory stimulus, induced by 14-days of daily LPS administration (*n* = 3 in each cohort). Steady SpO2 values impugn the prospect of LPS-induced impairments to pulmonary blood oxygenation

LPS was applied daily for 14 days to assess how systemic inflammation affects cerebral oxygenation and hemodynamics in AD and wildtype mice. Figure 3 illustrates how arterial and venous SO2 changes in diving arterioles and ascending venules over the course of the inflammatory threat. The same vessels were measured before, during and after induced inflammation (Fig. 3A). We averaged the measurements over animals to obtain the mean pO2 & SO2 in the two cohorts. Intriguingly, while systemic inflammation reduced microvascular oxygen levels in both cohorts, the effects were more pronounced and occurred earlier in AD mice (Fig. 3B-C, Additional file 1: Supplementary Fig. 1B-C). Within 7 days, LPS-induced inflammation substantially decreased arteriole and venule SO2 at all cortical layers in AD mice. By contrast, only mild, nonsignificant reductions were observed in WT mice at 7 days. In both cohorts, the corresponding changes to SO2 were most pronounced in ascending venules (Fig. 3B-C). The inflammatory threat induced substantial increases in computed depth-dependent oxygen extraction (DOE = SaO2 – SvO2) for AD mice in all cortical layers at 7 days of LPS administration (Fig. 3D). Conversely, in WT mice, significant increases in DOE were observed only in layer I at 7 days (Fig. 3D). At 14 days of LPS injection, both cohorts exhibited slight recovery in vascular oxygenation and oxygen extraction.

### LPS-induced inflammation does not impair pulmonary gas exchange

In AD mice, the significant oxygen reductions in O2-supplying arterioles at all cortical layers were unanticipated (Fig. 3B, and Additional file 1: Supplementary Fig. 1B). Prior studies in awake healthy mice indicate that only modest amounts of oxygen are delivered from cortical arterioles [44, 62, 65]. Consequently, a reduction in this upstream vascular compartment warranted additional investigation. To determine whether the diminished oxygen in cortical arterioles results from impaired pulmonary gas exchange, we monitored the systemic arterial oxygenation (SpO2) in a separate group of AD and WT mice during 14 days of induced systemic inflammation. Insufficient oxygen uptake in the lung compartment would yield global reductions in oxygen supply and therefore cause substantially lower SpO2. Our daily measurements from the mouse thigh revealed no appreciable changes of SpO2 in either mouse cohort during the course of a 14-day inflammatory threat (Fig. 3E), which belies the possibility of diminished pulmonary gas exchange induced by systemic inflammation.

### LPS-induced inflammation decreases capillary pO2 but not capillary RBC flux

**Fig. 4:**
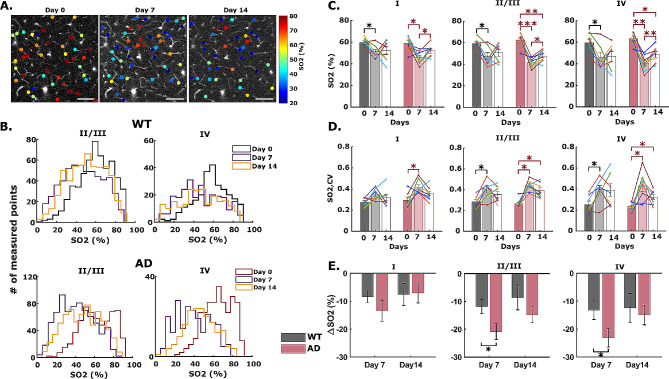
Inflammation-induced cerebral capillary oxygen reduction in WT and AD mice brain. (**A**) Example survey scan images overlaid with SO2 values in measured capillary segments at z = 200 μm taken on day 0 (before LPS injection), day 7 and day 14 (with continuous LPS injection) inside an AD mouse brain. Scale bar = 100 μm. (**B**) Histogram showing layer-specific SO2 distributions in all measured capillary points from all animal samples in both cohorts. (**C-D**) Capillary SO2 and the corresponding coefficient of variation (CV) at baseline (day 0), and with LPS-induced inflammation (day 7 and 14) in WT and AD mice brain in cortical layer I, II/III, and IV. Each bar represents mean ± standard error over all measured mice in each cohort. Connected scatterplot shows the SO2 of each individual mouse. (**E**) Absolute changes in capillary SO2 in WT and AD mice with 7 and 14 days of LPS injection. ΔSO2 is calculated by subtracting the SO2 on day 0 from the SO2 on day 7 or 14

Capillary pO2 measurements were performed in the same fields of view on days 0, 7 and 14 of LPS-induced inflammation (Additional file 1: Supplementary Fig. 2), and corresponding SO2 values were calculated using the Hill equation. Figure 4 displays how LPS-induced inflammation alters capillary SO2 and their associated coefficients of variation (CV) at different cortical layers. After 7 days, notable capillary SO2 decreases were observed in all cortical layers of WT brain (Fig. 4B-C). In AD mice, the inflammatory threat yielded more drastic reductions (Fig. 4E). In both AD and WT cohorts, the most pronounced oxygen reductions were observed in cortical layer IV (Fig. 4E, ΔSO2 = − 13.22 ± 3.36% in WT and ΔSO2 = − 23.17 ± 3.28% in AD). Capillary oxygenation became substantially more heterogenous in both cohorts under inflammatory threat, as indicated by the increased CVs of capillary SO2 (Fig. 4D). Prior modeling studies indicate that greater dispersity of capillary transit time markedly limits total O2 extraction from blood [44, 70, 74]. Guided by their findings, our pO2 and SO2 heterogeneity observations strongly indicate that systemic inflammation substantially disrupts the delicate balance between oxygen supply and demand in cortical brain tissue.

Interestingly, after 14 days of LPS injection, cerebral capillary oxygenation and oxygen heterogeneity demonstrated a slight tendency to recover in both mice cohorts (Fig. 4C-D, and Additional file 1: Supplementary Fig. 2). This observation motivated additional investigations into the duration of inflammatory disruptions. In a subset of the remaining animals (*n* = 2 AD, *n* = 2 WT), arteriolar, venular, and capillary oxygen measurements recovered to the baseline level after 70 days (∼ 10 weeks) since the final LPS injection (Additional file 1: Supplementary Fig. 3). Additional investigations will explore the extent of reversibility of inflammation’s metabolic disruptions.

**Fig. 5:**
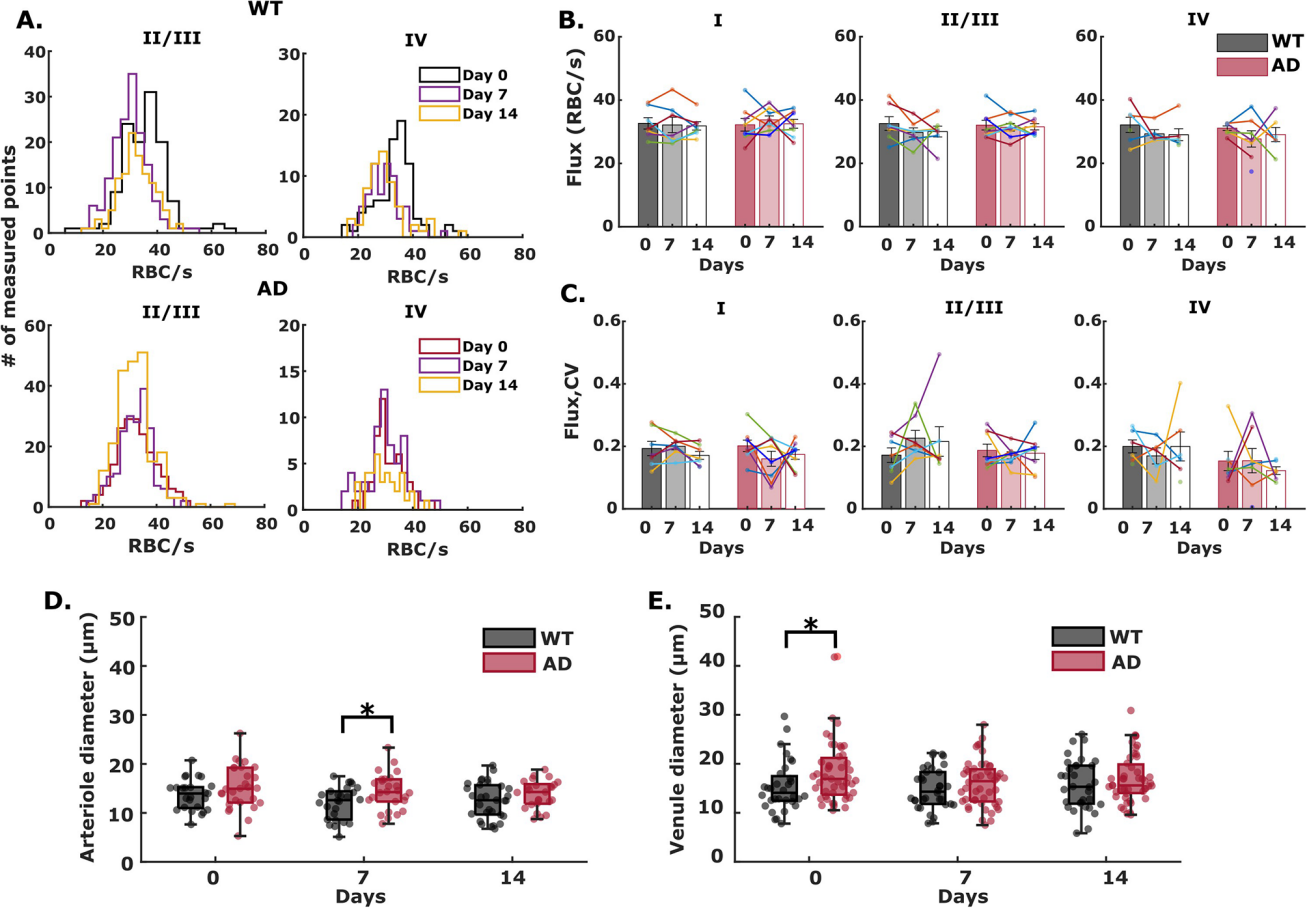
Cerebral capillary red blood cell flux (RBC flux) at day 0 (no LPS injection), day 7 and day 14 (with LPS injection) in WT and AD brain. (**A**) Histograms showing RBC flux in all measured capillary points in cortical layers II to IV. (**B-C**) Mean RBC flux and coefficient of variation over all measured mice in both cohorts over the course of LPS-induced inflammation. Each bar represents mean ± standard error over all measured mice in each cohort. Connected scatterplot shows the RBC flux and the CV of each individual mouse. **D-E** Diameter of penetrating arteriole and venule in WT and AD mice on day 0, day 7 and day 14

In contrast to its effects on intravascular oxygenation, systemic inflammation did not significantly affect layer-dependent capillary RBC flux in AD or WT mice (Fig. 5). In WT mice, we observed mild, but not significant reductions in RBC flux within 7 days in layers II – IV. The changes were even less pronounced in AD mice (Fig. 5A-B). On average, the coefficients of variation for RBC flux did not change appreciably after systemic inflammation (Fig. 5C), suggesting that spatial blood flow heterogeneity remains largely unaffected by an inflammatory threat in 8-month-old mice.

To further assess potential alterations in cerebral blood supply induces by LPS, we calculated the diameter of the measured penetrating arterioles and venules in layer I using the 2D raster scan images. Our result shows that a two-week LPS injection does not induce changes in arteriole and venule diameter (Fig. 5D, E). The maintained vessel diameter and RBC flux after LPS suggested no changes in cerebral blood volume and thus no increase in O2 supply.

### LPS induces inflammatory secretions in microglia and astrocytes

**Fig. 6:**
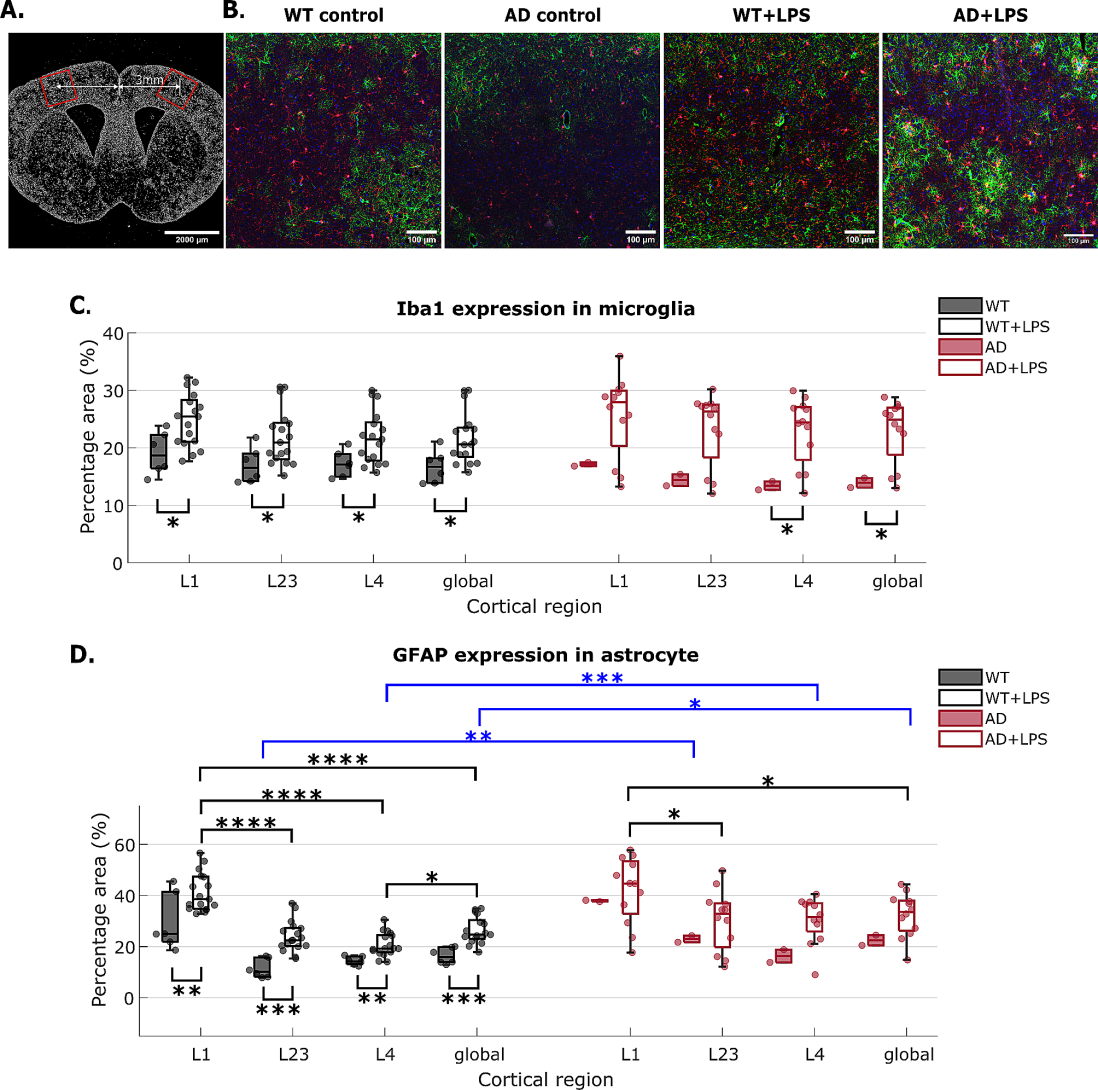
Immunofluorescence analysis of LPS’s effect on microglia and astrocyte activation in WT and AD mice. (**A**) Example coronal brain sections with the imaged field of view (around somatosensory cortex) indicated in red rectangle boxes. Image was captured with confocal tile imaging using 5X objective. Fluorescence signal is DAPI-labelled nuclei. (**B**) Example maximum intensity projection (MIP) of confocal images of WT and AD mice. Control groups are mice that did not receive LPS injection (*n* = 1 in each cohort). Green: astrocyte. Red: microglia. Blue: nuclei. Images displayed have the same intensity threshold. (**C**) Percentage area (PA) of Iba1 positive microglia and (**D**) GFAP positive astrocyte in different cortical layers and the entire imaged region of interest (ROI) around somatosensory cortex (denoted as ‘global’). Each mouse has two to six ROIs imaged from two to three micro-thickness brain slices. Filled black and red dots represent the result of a single ROI. Statistical difference between layers were tested using one-way ANOVA and Tukey’s post-hoc test. Difference between WT and AD was revealed by student’s t-test

Glial fibrillary acidic protein (GFAP) and ionized calcium-binding adaptor molecule (Iba-1) are biomarkers of microglia and astrocytes, respectively, and are upregulated in activated microglia and astrocytes during neuroinflammation [75, 76]. We quantified the cortical-layer specific activation of microglia and astrocytes through immunofluorescence staining of Iba1 and GFAP in AD and WT mice after 14 days of daily LPS administration. Somatosensory cortex in both hemispheres were imaged with confocal microscopy (Fig. 6A, B). Prior to inflammation, there were no significant differences in the expression of Iba1 between WT and AD mice (Fig. 6C). AD mouse has significant higher expression of GFAP in cortical layer II/III but not the overall cortical layer I-IV (Fig. 6D), consistent with previous studies showing modest, nonsignificant inflammation in 6-month-old APP/PS1dE9 mice cortex [77, 78].

Under 14 days of LPS-induced inflammation, we observed elevated expression of microglial Iba1 and astrocytic GFAP in both WT and AD mice (Fig. 6C, D). In WT mice, LPS significantly increase the microglial Iba1 and astrocytic GFAP in all cortical layers. In AD mice, LPS significantly increases microglial Iba1 expression in cortical layer IV but not in layer I-III, suggesting a layer-dependent activation of microglia under LPS. The elevation of astrocytic expression of GFAP is not statistically significant in AD mice. WT and AD group has no significant difference in terms of Iba1 expression, however, the expression of GFAP in AD was significantly higher than WT, reaching statistical significance in cortical layer IV and the global imaged cortical area (Fig. 6D), suggesting a higher extent of inflammation in AD mice brain under the effect of LPS.

## Discussion

Neuroinflammation reportedly has both therapeutic and harmful effects during AD progression [1, 40]. The conflicting influences of neuroinflammation have motivated several studies exploring the effects of modulating neuroimmunity [41]. The impact of a pronounced inflammatory threat on cerebral energetics and microvascular hemodynamics warrants much further investigation to guide effective diagnostic and therapeutic developments. To our knowledge, we provide the first quantitative measurements of microvascular oxygen pressure, oxygen extraction, and RBC flux in the APP/PS1dE9 mouse cortex before, during, and after a pro-inflammatory stimulus.

Under baseline conditions (in the absence of inflammatory stimulus), our results in WT mice indicate that depth-dependent oxygen extraction (DOE) increases along cortical layers, consistent with previous 2P-PLIM measurements in awake C57 mice (Fig. 2) [44, 69]. AD mice have slightly higher pO2 and oxygen saturation in arterioles, capillaries, and venules at all cortical layers, corresponding to progressively lower DOE at deeper cortical layers. Conversely, no significant differences in capillary RBC flux were found in either AD or wildtype cohorts at any cortical layer. Taken together, these observations suggest that early amyloid accumulation induces reductions in cortical oxygen extraction without yet substantially altering vascular oxygen supply in 8-month-old APPswe/PS1dE9 mice. Our results suggest that cerebral metabolic rate of oxygen (CMRO2 = CBF * OEF) would be reduced in AD mice, primarily due to reductions of the brain’s metabolic demand.

Our baseline observations in AD mice agree well with human MRI-studies in patients with amnestic mild cognitive impairment (MCI, regarded as an early stage of AD) [79]. Global cerebral blood flow was found to be similar in MCI patients relative to age-matched control patients, while OEF was lower in MCI patients by ∼ 10%. Consequently, with no notable changes to blood supply, the authors attributed the ∼ 12.5% lower global CMRO2 in MCI patients to a reduction in metabolic demand by the brain tissue. Also consistent with our results, the same authors reported that OEF was reduced in later-stage AD patients, and the magnitude of OEF reduction correlated well with severity of cognitive impairment [80].

In our results, the significantly lower DOE in cortical layer IV in AD mice indicates lower demand of oxygen by layer IV neurons, which is likely the result of reduced neural activity. This correlates well with observations of reduced mitochondrial function in AD mice at age 7–10 months [81] and reduced EEG rhythms observed in MCI patients [79]. The lower DOE in our 8-9-month-old AD mice are likely a consequence of Aβ-induced impairments to mitochondrial function and diminished neuronal activity [81–83]. Contrast-enhanced MRI measurements in APPswe/PS1dE9 mice showed no changes in relative cerebral blood volume (rCBV) at 8-month-old mice but significant reduction at 15-month-old mice [84]. We expect that APPswe mice would likely also experience pronounced impairments to CBF at more advanced ages that exhibit more severe amyloid pathology. Future investigations will explore how oxygen extraction and other metabolic markers vary at more advanced ages in AD mice, as well as age-dependent susceptibility to systemic inflammatory threats.

While our observations indicate that baseline oxygen extraction in AD mice is lower than WT mice, an inflammatory insult provoked significant reduction in blood oxygen in both animal groups. More pronounced changes to SO2 and DOE were consistently observed in AD mice (Figs. 3 and 4). Conversely, we found no significant inflammation-induced changes to arterial and venous diameters or capillary RBC flux in cortical layers I-IV (Fig. 5), indicating that blood volume and blood flow remain unaffected by neuroinflammation at age 8 months.

It is noteworthy that the LPS-induced reduction of vascular pO2 appears in all cortical layers, including layer I. Compared with deeper layers, layer I contains fewer neuronal cell bodies but higher number of excitatory and inhibitory synapses [85]. The observed reductions in layer I vascular pO2 indicate increased metabolic demand. Previous study showed that LPS-induced systemic inflammation increases the frequency of spontaneous Ca^2+^ transient in axons at layer I of mouse frontal/motor cortex [86]. LPS-induced cytokine release, such as TNF-α, was shown to increase excitatory synaptic strength while decreasing inhibitory synaptic strength [87]. Consequently, we hypothesize that increased excitatory synaptic activity accounts for LPS-induce increases in metabolism within layer 1.

Our results indicate that neuroinflammation potentially alters cerebral oxygen demand but not supply. We posit that the increased oxygen extraction from the vascular compartments is the net result of multiple pathological processes triggered by the inflammatory insult. Increased metabolic demand by hyperactive neurons most likely accounts for the majority of increased O2 consumption during inflammation [86, 88]. However, in addition to increased energetic demand, oxygen extraction from the blood is also likely enhanced by increased permeability across the blood brain barrier (BBB) and the multifaceted actions of immune cells. Numerous studies have demonstrated that LPS administration yields dramatic increases in spontaneous calcium spiking and excitatory post-synaptic currents from neurons in the cortex, hippocampus, and several other regions of the brain, while also reducing the activity of inhibitory GABAergic interneurons [55, 86, 89–94]. Because restoration and maintenance of ion gradients accounts for the bulk of ATP consumption in the brain, and energy consumption increases markedly with increased action potential frequency, inflammation-induced neuronal hyperactivity across different brain regions constitutes a massive additional energetic burden [95].

Neuronal hyperactivity likely accounts for the majority of increased oxygen demand during neuroinflammation; however, inflammation’s influence on the BBB could also contribute to increased oxygen extraction from the vascular compartment [88]. LPS notably disrupts multiple structural and functional components of the BBB, including the tight junctions linking endothelial cells, morphology and function of astrocytes, and expression of the efflux transporter protein permeability-glycoprotein (P-gp), and vessel surface area [96–99]. Each of these alterations substantially augments BBB permeability and efflux of cytokines and small molecules. These inflammatory reductions in structural and functional integrity of the BBB likely further promote passive diffusion of oxygen across the vessel walls, particularly in the arterial vessels.

In addition to neuronal hyperexcitability and BBB disruption, neuroinflammation also triggers a host of actions by immune cells with varied effects on cerebral oxygen demand. Upon activating to their pro-inflammatory phenotype to combat pathological threats, microglia and astrocytes avidly produce inflammatory cytokines, increase phagocytic activity, and undergo morphological changes to more ameboid profiles. To facilitate cell proliferation and rapid cytokine production, activated microglia reportedly shift their metabolism to rely more on glycolysis, while also experiencing increased mitochondrial fission [30, 33, 52]. While the former adaptation reduces microglial oxygen dependence for metabolism and decreases intracellular pH [100], investigators observed that LPS exposure actually increases oxygen consumption rate (OCR) in microglia, particularly OCR associated with mitochondrial proton leak [101]. Despite their purported shift to glycolytic metabolism, our observations support prior studies, which demonstrate that activated microglia consume higher amounts of oxygen to mediate ROS production to neutralize pathogenic threats. Microglia show similar metabolic behavior to peripheral immune cells such as monocytes, leukocytes, and neutrophils, which also contribute to the neuroinflammation response and can increase ROS generation by 50-fold during high immune activity [28, 30, 102–104].

Overall, our data support prior findings indicating that increases in BBB permeability and increased oxygen demand from hyperactive neurons and pro-inflammatory immune cells is substantial enough to extract oxygen from the arteriolar compartment. In contrast to the classical Krogh model of exclusive oxygen extraction from the capillary compartment, significant oxygen extraction from arterioles has been observed previously in healthy mice under isoflurane anesthesia. In the absence of anesthesia, though, similar measurements in awake mice agree better with the Krogh model [44, 46, 65, 69]. Our observations indicate that pronounced neuroinflammation substantially, but reversibly, shifts the balance of metabolite supply and demand in the brain.

Compared to wildtype mice, the more aggravated response in AD mice can be attributed to a deteriorative cycle of cytokine release and impaired mitochondrial function, initiated and promoted by continued accumulation of cytotoxic amyloid β. A prior study showed that APP/PS1dE9 mice at age 9–12 months secrete significantly higher TNF and IL-1β than wildtype mice in response to long-term systemic LPS injection [105]. Similarly, our IHC results indicate that LPS triggers greater release of IBA-1 and GFAP from AD mice, particularly in cortical layer IV (Fig. 6D), supporting the assertion that AD mice favor proinflammatory phenotype upon innate immune stimulus. Prior reports in both humans and mice also demonstrate links between elevated levels of proinflammatory cytokines (e.g., TNF-α) and increased both tau and cytotoxic amyloid pathology [106–108]. In essence, a positive feedback loop exists between amyloid pathology and neuroinflammation that further exacerbates the brain’s pathological disruptions. Energy metabolism and oxygen demand contribute to this destructive feedback loop through inflammatory signaling’s marked impairments to mitochondrial function. Cytokines such as TNFα and IL-1β as well as ROS disrupt mitochondrial enzyme activity involved in the TCA cycle, inhibits oxidative phosphorylation (OXPHOS), and reduces ATP production [20]. LPS reportedly induces both short- and long- term reductions on OXPHOS by downregulating activity of complexes I, II, and IV. While the exact mechanisms are not precisely understood, one proposed model hypothesizes that the LPS-induced torrent of cytokines such as TNFα activate a downstream tyrosine kinase, leading to the phosphorylation of complex IV and strong inhibition of all electron transport chain activity. Ultimately, impairments to the TCA cycle and electron transport chain significantly reduces mitochondrial membrane potential and efficiency of oxidative phosphorylation [109]. Leaked components from dysfunctional mitochondria could in turn further activate innate inflammatory response and impair Aβ clearance [110]. A vicious cycle ensues between Aβ, inflammation, mitochondrial dysfunction, and oxidative stress in AD mice brain that lower the efficiency of oxygen usage and ATP synthesis. As a result, tissue oxygen extraction is greatly increased to sustain the heightened energy needs and inflammatory response in the AD brain (Fig. 3).

In both cohorts, induced inflammation did not significantly affect cerebral capillary RBC flux (Fig. 5). Our results are consistent with previous reports of no change of cerebral blood flow after LPS-induced acute inflammation [111, 112]. Fruekilde et al. reported increased resting-state capillary stalling under LPS-induced acute inflammation due to leukocyte plugging [113].

## Conclusions

This study demonstrates that induced systemic inflammation alters cortical oxygen demand but not oxygen supply in a mouse model of Alzheimer’s disease at the age of 8–9 months. Our findings substantiate purported links between neuroinflammation and energy metabolism in preclinical AD pathology. The results will help advance the understanding of the critical role inflammation plays in Alzheimer’s disease and the utility of oxygen extraction fraction (OEF) in AD diagnosis. Our preclinical investigation provides insight to better interpret observations from translational neuroimaging using modalities such as functional magnetic resonance imaging (fMRI) and positron emission tomography (PET). And the interaction between AD brain immune response and cerebral oxygen metabolism should be taken into account when developing AD treatment targeting neuroinflammation.

One limitation of our study is that we did not label Aβ. Further investigations will explore longitudinal labeling of Aβ during systemic inflammation to correlate the oxygenation changes with amyloid pathology. An additional potential limitation exists in our calculations of cortical SO2, which did not account for possible inflammatory changes to the oxygen dissociation curve. The conventional Hill equation utilizes constant, physiological values to relate SO2 and pO2. The Hill equation does not account for inflammatory changes to blood pH, temperature, or other influences like concentrations of CO2 and 2,3 diphosphoglycerate. Modeling studies demonstrate that each of these factors induces significant shifts to the oxygen dissociation curve in human blood [114, 115]. Experimental validation is still required to determine the full effects of LPS-induced inflammation in blood of APPswe/PS1dE9 mice. LPS reportedly lowers intracellular pH but not extracellular pH in brain slice experiments [100], and our oximetry measurements of systemic SO2 were unaffected during 14 days of LPS treatment (Fig. 3. E). These results suggest that our potential inflammatory pH and temperature changes do not substantially alter the oxygen dissociation curve.

Though we observed no significant differences in baseline capillary RBC flux and spatial heterogeneity, our results do not comprehensively refute prospective impairments to CBF and microvascular hemodynamics at early stages of AD. Our observations are consistent with a prior study of capillary RBC flux in the barrel cortex of 6-month APP/PS1dE9 mice [71]. Hypoperfusion is widely recognized as an early pathological indicator of AD, but conflicting studies with different animal models, different ages, and different measurement techniques confound a detailed understanding of the precise timing, severity, and spatial heterogeneity of CBF impairments during the preclinical stage of AD [12, 34, 71, 116]. Some studies showed no obvious changes in CBF and capillary flow in young mice but severe reduction of flow and disturbed flow homogenization in aged transgenic mice [73]. Recent advances in optical coherence tomography (OCT) will help delineate the precise trajectory of hemodynamic alterations during AD by enabling near-lifespan tracking of microvascular morphology and function [117]. Lastly, estimating cerebral metabolic of oxygen (CMRO2) in response to inflammation using Krogh-Erlang model [118] or the recent proposed ODACITI model [119] could further validate our findings of increased oxygen extraction in inflamed AD brain tissue.

### Electronic supplementary material

Below is the link to the electronic supplementary material.

Supplementary Material 1

## Data Availability

Data generated and analyzed in the study are available upon request.

## Abbreviations

2P-PLIM: Two-photon phosphorescence lifetime imaging microscopy
AD: Alzheimer’s disease
APP: Amyloid precursor protein
ATP: Adenosine triphosphate
Aβ: amyloid-beta
CBF: Cerebral blood flow
CMRO2: Cerebral metabolic rate of oxygen
CV: Coefficient of variation
DOE: Depth-dependent oxygen extraction
fMRI: Functional magnetic resonance imaging
GFAP: Glial fibrillary acidic protein
IBA-1: Ionized calcium-binding adaptor molecule 1
LPS: Lipopolysaccharide
NGVU: Neuro-glio-vascular unit
OCT: Optical coherence tomography
PET: Positron emission tomography
pO2: Partial pressure of oxygen
PS1dE9: Presenilin 1 gene
RBC: Red blood cell
ROS: Reactive oxidative species
SO2: Oxygen saturation
TCA: Tricarboxylic acid cycle
