# Supplementary material for: Neuroinflammation increases oxygen extraction in a mouse model of Alzheimer’s disease
Source: Alzheimers Res Ther. 2024 Apr 10;16:78. doi: 10.1186/s13195-024-01444-5 (PMC11005245; doi:10.1186/s13195-024-01444-5)
Supplement: Supplementary file 1 — Supplementary Material 1 [file 13195_2024_1444_MOESM1_ESM.docx]

Supplementary figures for

Neuroinflammation increases oxygen extraction in a mouse model of Alzheimer’s disease

Chang Liu^1^, Alfredo Cárdenas-Rivera^1^, Shayna Teitelbaum^1^, Austin Birmingham^1^, Mohammed Alfadhel^1^, Mohammad A. Yaseen^1^

1 Department of Bioengineering, Northeastern University, Boston, MA 02115, USA

**Supplementary Table 1. Number of arterioles, capillaries, venules and number of animals (n) measured for pO2.**

| **Cohort** | | **WT** | | | **AD** | | |
| --- | --- | --- | --- | --- | --- | --- | --- |
| **Measurement** | **Vessel type** | **Layer**  **I** | **Layer II/III** | **Layer**  **IV** | **Layer**  **I** | **Layer**  **II/III** | **Layer**  **IV** |
| **Day 0**  **(Baseline)** | **arterioles** | 22 (10) | 21 (9) | 15 (7) | 18 (9) | 18 (9) | 8 (6) |
|  | **venules** | 43 (10) | 43 (10) | 29 (6) | 44 (9) | 44 (10) | 28 (8) |
|  | **capillaries** | 461(7) | 549(7) | 252(7) | 497 (8) | 606 (8) | 269 (8) |
| **Day 7** | **arterioles** | 13 (7) | 10 (7) | 6 (5) | 16 (8) | 15 (8) | 6 (5) |
|  | **venules** | 27 (7) | 27 (7) | 8 (4) | 37 (8) | 35 (8) | 15 (7) |
|  | **capillaries** | 512(7) | 548(7) | 247(7) | 701(8) | 735 (8) | 223 (8) |
| **Day 14** | **arterioles** | 16 (7) | 14 (7) | 6 (5) | 16 (8) | 14 (8) | 5 (5) |
|  | **venules** | 30 (7) | 29 (7) | 8 (4) | 37 (8) | 33 (8) | 17 (7) |
|  | **capillaries** | 537(7) | 551(7) | 218(7) | 609 (8) | 695 (8) | 179 (8) |

**Supplementary Table 2. Number of capillaries and animals (n) measured for RBC flux.**

| **Cohort** | **WT** | | | **AD** | | |
| --- | --- | --- | --- | --- | --- | --- |
| **Measurement** | **Layer I** | **Layer II/III** | **Layer IV** | **Layer I** | **Layer II/III** | **Layer IV** |
| **Day 0 (Baseline)** | 104 (7) | 122 (7) | 79 (6) | 162 (8) | 191 (8) | 42 (6) |
| **Day 7** | 146 (7) | 156 (7) | 66 (6) | 163 (8) | 200 (8) | 60 (6) |
| **Day 14** | 147 (7) | 116 (7) | 60 (6) | 145 (8) | 219 (8) | 33 (6) |


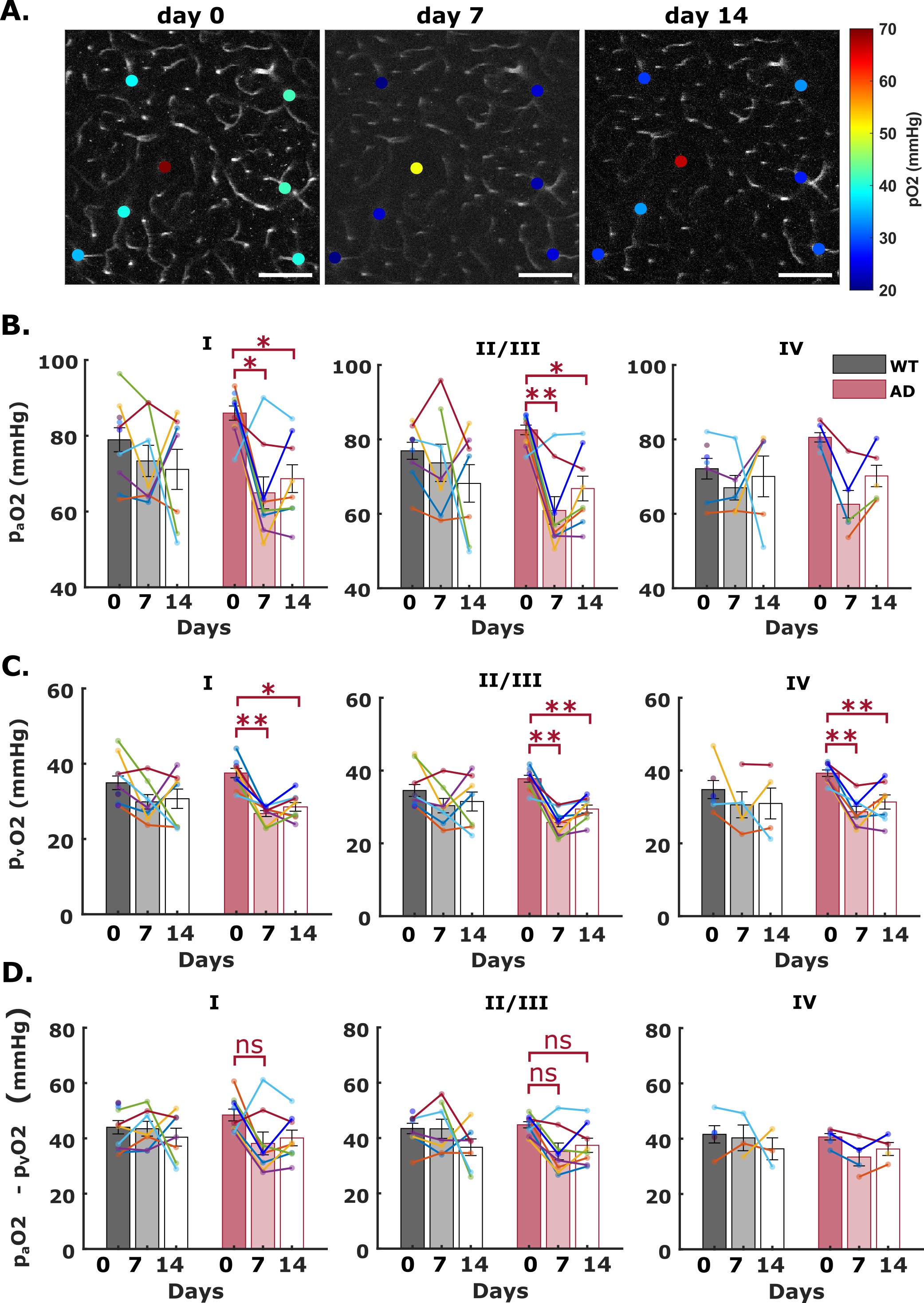


**Supplementary Fig. 1 Inflammation-induced arteriole and venule pO2 reductions in WT and AD mice brain.** **(A)** Example survey scan images overlaid with pO2 values in measured penetrating vessels at z=200 µm taken on day 0 (before LPS injection), day 7 and day 14 (with continuous LPS injection). Scale bar = 100 µm. **(B-C)** Arteriole and venule pO2 measured on day 0, and day 7 and day 14 in WT and AD mice at cortical layer I, II/III, and IV. Each bar represents mean ± sem over all measured mice in each cohort. Connected scatterplot shows the pO2 of each individual mouse. **(D)** pO2 difference between penetrating arteriole and ascending venules in cortical layer I to IV.


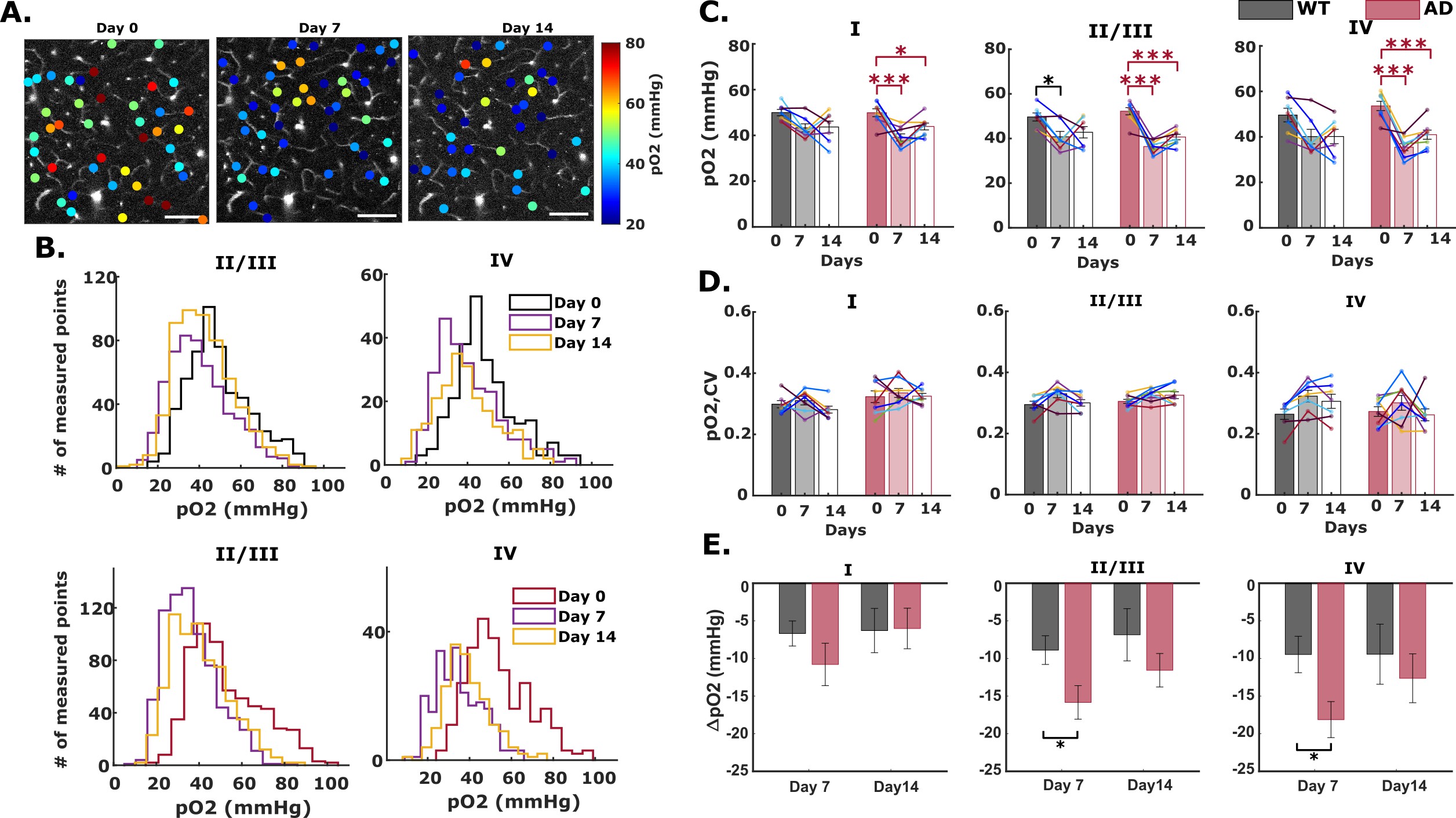


**Supplementary Fig. 2 Inflammation-induced cerebral capillary oxygen reduction in WT and AD mice brain.** **(A)** Example survey scan images overlaid with pO2 values in measured capillary segments at z=200 µm taken on day 0 (before LPS injection), day 7 and day 14 (with continuous LPS injection). Scale bar = 100um. **(B)** Histogram showing layer-specific pO2 distributions in all measured capillaries at cortical layer II to IV. **(C-D)** Capillary pO2 and the corresponding coefficient of variation (CV) at baseline (day 0), and with LPS-induced inflammation (day 7 and 14) in WT and AD mice brain at cortical layer I, II/III, and IV. Each bar represents mean ± standard error over all measured mice in each cohort. The connected scatterplot shows the pO2 of each individual mouse. **(E)** Absolute changes in capillary pO2 in WT and AD mice with 7 and 14 days of LPS injection


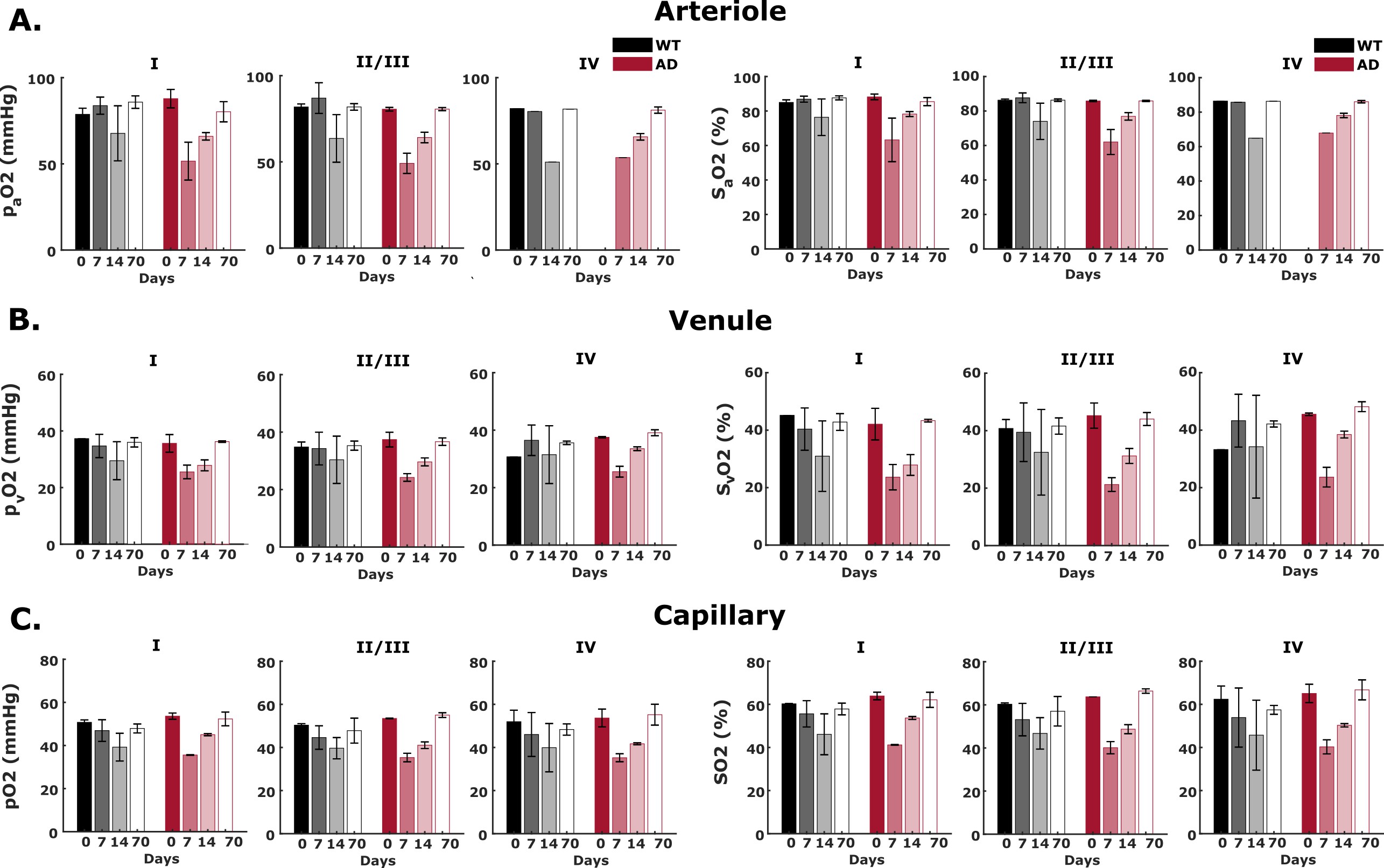


**Supplementary Fig. 3 Recovery of cerebral intravascular oxygenation in WT and AD mice (n=2 in each cohort).** pO2 and SO2 in arterioles **(A)**, venules **(B)**, and capillaries **(C)** were measured 70 days after the last LPS injection. pO2 and SO2 in all three vessel types in the measured mice were recovered to the same level as baseline (before LPS-induced inflammation). Data displayed as the mean pO2 (SO2) in cerebral arteriole, venules, and capillaries over two AD and two WT mice. Error bar represents the standard error
